# Ductal Carcinoma Arising in a Squamous Epithelial Inclusion Cyst within an Axillary Lymph Node: A Challenging Nodal Metastasis

**DOI:** 10.1155/2023/9979532

**Published:** 2023-10-31

**Authors:** Kaitlyn J. Nielson, Ruifeng Guo, Malvika H. Solanki, Charles D. Sturgis

**Affiliations:** ^1^Department of Laboratory Medicine and Pathology, Mayo Clinic, Rochester, Minnesota, USA; ^2^Department of Laboratory Medicine and Pathology, Mayo Clinic, Jacksonville, Florida, USA

## Abstract

*Introduction*. Assessment of axillary lymph nodes in breast carcinoma is an important part of staging to guide appropriate clinical management. Lymph node inclusions of different types, including nevoid, squamous, and glandular, are rare but have been reported in multiple different anatomic locations including the axilla. These can result in diagnostic challenges and pose risks of misdiagnoses. Rarely, malignancies may arise intrinsic to otherwise incidental benign nodal inclusions. *Case Presentation*. We report a case of ductal carcinoma diagnosed within a squamous epithelial inclusion cyst within an axillary lymph node in a patient with pure ductal carcinoma in situ (DCIS) of the ipsilateral right breast. To our knowledge, this is the fifth report in the literature of breast carcinoma confirmed within an axillary inclusion in a patient with pure DCIS. Evaluation of the primary DCIS and lymph node inclusions, by routine and immunohistochemical stains, was performed for assessment. *Discussion*. The presence of lymph node inclusions can pose a challenge in assessment of benignity and malignancy, on frozen and permanent histologic sections. Pathologists should carefully evaluate lymph node inclusions to ensure that intrinsic malignancies are not missed within rare otherwise benign appearing incidental epithelial rests.

## 1. Introduction

Histologic assessment of axillary lymph node status is one of the most important parameters for determining prognosis for patients with early-stage breast carcinomas. The utilization of sentinel lymph node biopsies to assess nodal status has become standard of care to determine the need for further axillary dissection and adjuvant therapies in a way that limits morbidity in comparison to the previously performed axillary lymph node dissection [1]. The use of sentinel lymph node biopsy in patients diagnosed with ductal carcinoma in situ (DCIS) without evidence of invasion is variable and depends upon the individual patient's clinical setting, the grade and extent of the DCIS, and practice patterns in different centers. The incidence of sentinel lymph node metastases in patients with DCIS alone has been reported to range from 1 to 13%, meta-analyses confirming <5% of patients with DCIS to ultimately have nodal disease. Those who are confirmed to have nodal disease are most often patients with large volume DCIS (>5 cm or palpable) and/or suspicion of microinvasion in their breast histology [2, 3].

Sentinel lymph node biopsy histologic evaluation can be challenging, with subtle metastatic disease being difficult to separate from various benign mimics. Axillary lymph node inclusions, for example, are one such challenge. Lymph node inclusions may be glandular, squamous, or nonepithelial in nature and should be carefully assessed to properly stage breast cancer [4]. Regarding axillary nodes, a number of inclusions have been reported, including heterotopic breast parenchyma including papillomas with proliferation, endosalpingiosis, squamous nests/cysts, and nevi [4–7]. While these inclusions harbor no adverse effects, it is important to recognize benign axillary lymph node inclusions and study them carefully, as malignancy can rarely be identified intrinsic to epithelial inclusions in lymph nodes of patients with ipsilateral breast carcinomas [6–9].

We herein describe a case of ductal carcinoma within an epithelial inclusion in an axillary sentinel lymph node in a patient with pure DCIS.

## 2. Case Presentation

The patient was a 66-year-old female, with no reported personal or family history of breast or related carcinomas, who presented for routine breast cancer screening at an outside center. She underwent screening and subsequent diagnostic mammography, which identified three clusters of abnormal calcifications in the upper outer quadrant of her right breast. Core biopsy was performed at the outside facility, and a diagnosis of high-grade ductal carcinoma in situ (DCIS), estrogen receptor (ER) negative, was rendered. Progesterone receptor (PR) status was not available for review. Additional magnetic resonance imaging (MRI) performed at the outside facility reported a large area of suspicion in the right breast, as well as additional suspicious areas in the left breast; therefore, the patient proceeded to bilateral mastectomy and right sentinel lymph node biopsy. Per report, the right mastectomy confirmed high-grade DCIS with no evidence of invasion. Histologic assessment at the outside facility raised a question regarding a cystic epithelial structure present in a sentinel lymph node. Representative slides were received at our institution for expert consultation. The majority of the patient's care was performed at the outside institution, and additional detailed clinical information regarding the patient and clinical care was not available for review.

**Figure 1:**
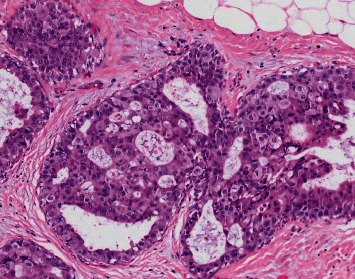
Photomicrograph from right mastectomy confirming ductal carcinoma in situ, high-grade, clinging, and cribriform patterns (hematoxylin and eosin (H&E) stain, 200x).

One representative slide from the right breast mastectomy was received from the outside institution. High-grade DCIS with clinging and cribriform growth patterns and associated necrosis was confirmed (Figure 1).

**Figure 2:**
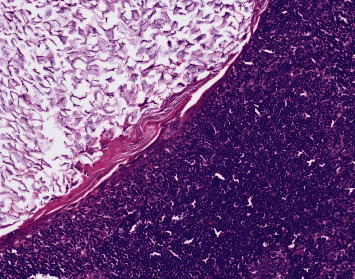
Photomicrograph of the patient's right axillary sentinel lymph node with epithelial inclusion cyst showing central keratinous debris and a bland squamoid lining (hematoxylin and eosin (H&E) stain, 200x).

Histologic assessment of the sentinel lymph node showed predominantly benign lymph node parenchyma with a cortical cystic structure containing keratinous debris. In most areas, this intranodal cystic structure was lined by bland, spindled to cuboidal squamoid-appearing cells (Figure 2).

**Figure 3:**
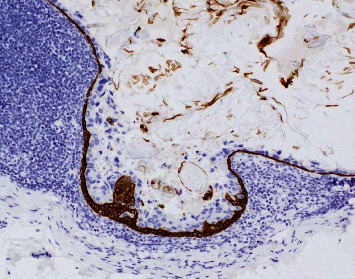
Photomicrograph of the patient's right axillary sentinel lymph node with epithelial inclusion cyst, highlighting positivity of peripheral cyst lining and central keratinous debris (cytokeratin 5/6 immunohistochemical study, 200x).

Immunohistochemical (IHC) studies performed at our institution showed that these benign appearing squamoid cells were positive for p40, p63, and cytokeratin 5/6 (Figure 3).

**Figure 4:**
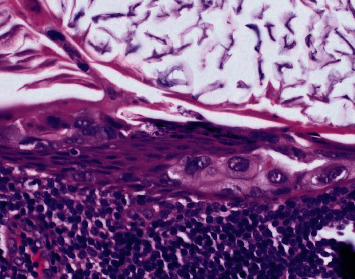
Photomicrograph of the patient's right axillary sentinel lymph node epithelial inclusion cyst with scattered groups of overtly malignant cells in the cyst lining that were morphologically identical to the patient's ipsilateral high-grade ductal carcinoma in situ (hematoxylin and eosin (H&E) stain, 600x).

Focal areas within this intranodal cystic structure showed thickening of the lining with larger, more pleomorphic cells with irregular nuclear membranes and large nuclei and nucleoli (Figure 4).

**Figure 5:**
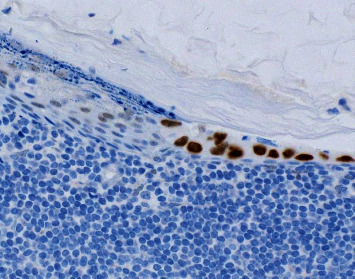
Photomicrograph of the patient's right axillary sentinel axillary lymph node with epithelial inclusion cyst and involvement of the inclusion cyst by cells of high nuclear grade ductal carcinoma (TRPS1 immunohistochemical stain, 200x).

These areas were negative by IHC for p40, p63, and cytokeratin 5/6. Instead, they were positive for keratin AE1/AE3, cytokeratin 7, GATA3, CEA, and trichorhinophalangeal syndrome type 1 (TRPS1) (Figure 5).

They were negative for ER. These malignant cells were morphologically very similar to the DCIS cells in the ipsilateral breast. The overall size of this area was between 1.5 mm and 2 mm (less than 2 mm). After review by multiple breast pathologists and a dermatopathologist, the nodal disease was classified as metastatic ductal carcinoma of the breast involving a cortical epidermal inclusion cyst of a sentinel lymph node and, based on size, designated as a micrometastasis. The patient's final pathologic stage was therefore classified as pTis pN1mi.

## 3. Discussion

While uncommon, epithelial and nevoid lymph node inclusions have been well reported in pelvic, abdominal, mediastinal, and axillary regions. Documentation of these findings is increasing as sentinel lymph node assessment in breast cancer continues to be standard of care, with a recent study highlighting 18 cases of epithelial inclusions in axillary lymph nodes, 9 of which were identified in the sentinel lymph nodes [10, 11]. There are three general categories for epithelial inclusions, including glandular, squamous, and mixed glandular-squamous, where glandular inclusions can be subcategorized into mullerian and mammary types. The etiology of these inclusions is unclear. Squamous inclusions are less commonly reported in the axillary region versus the cervical region and often are mixed with focal glandular components [12]. Mullerian inclusions in the pelvic and abdominal regions, such as endosalpingiosis, have been associated with undiagnosed serous tumors and salpingitis [4]. Endosalpingiosis involving axillary nodes has been reported, but the mechanism of its development is not well understood [5]. Mammary-type epithelial inclusions in axillary lymph nodes have a few possible explanations, including iatrogenic displacement via manipulation or procedures, heterotopia/metaplasia, or as a form of embryonal maldevelopment, the latter of which is generally favored amongst authors on this topic [6, 9]. Inclusions can include ductal epithelium, with apocrine change, and papillomas are also reported, as well as proliferative changes such as usual ductal hyperplasia (UDH) [6, 11].

To our knowledge, there have previously been four cases of breast carcinoma intrinsic to axillary lymph node inclusions in a patient with DCIS only [6–9]. In 2007, Srinivasan et al. reported the first unprecedented case of DCIS in a lymph node with associated benign glandular inclusions, which was morphologically similar to the patient's mammary DCIS. Additionally, p63 and anti-smooth muscle myosin antibody (SMMS1) confirmed the presence of a myoepithelial layer in the DCIS in the lymph node inclusion. Prior to this report, Barsky et al. described a pattern of metastatic invasive carcinoma called revertant metastatic cancer, indicating a reversion from an invasive pattern to a DCIS-like pattern. In those cases, a basement membrane layer was reportedly present, but not a myoepithelial layer [9, 13].

Following Srinivasan et al., Jaffer et al. reported a case of DCIS developing in an axillary lymph node that was involved by intraductal papillomas, UDH, and atypical ductal hyperplasia (ADH), in a patient with a lumpectomy for DCIS 10 years prior [7]. They postulated that papillary lesions are more prone to displacement given their inherently friable nature. They additionally noted that displaced or heterotopic cells are subject to the same types of changes that can occur in breast epithelium, which can lead to proliferative and possibly malignant changes, and therefore categorized their case in question as DCIS arising in displaced or heterotopic glands, rather than true metastatic disease.

In 2013 and 2018, two additional cases of DCIS involving an axillary lymph node in patients with DCIS-only breast cancer were reported, one of which was a sentinel lymph node biopsy and the other was an incidentally identified axillary tail lymph node. Both reports describe DCIS similar in morphology to the breast mastectomy specimen involving mixed glandular-squamous nodal inclusions [6, 8].

## 4. Conclusions

Benign inclusions in the axillary nodes can and have commonly been mistaken for metastatic carcinoma, particularly during sentinel lymph node evaluations for breast cancer, both on frozen and permanent histology [10, 14]. The presence of squamous epithelium may raise the question of metaplastic carcinoma or squamous differentiation, similar to a case reported by Fraggetta and Vasquez [15]. In our case, the inclusion within the sentinel lymph node was squamous-lined only, with the only glandular component being the malignant mammary cells morphologically and immunophenotypically similar to the patient's breast disease. While the mechanism or metastasis in this case is not well understood, given these findings, the disease was deemed best classified and treated as a micrometastasis.

Axillary lymph node status remains one of the most important prognostic factors for breast cancer patients, and studies have shown decreased overall survival for macrometastases and micrometastases [16]. Sentinel lymph node biopsy can help patients with low-stage disease avoid a morbid axillary lymph node dissection, without significant impacts on survival [17]. The use of sentinel lymph node biopsy in patients with in situ carcinoma only on biopsy is variable but often performed in patients with large volume disease, when there is a suspected risk of upstaging to invasive carcinoma [3]. In our case, while only DCIS was identified on mastectomy, it was extensive, which is most often the situation in which nodal disease is identified in patients with pure DCIS. Additional study is needed to determine the prognostic implications of nodal malignancies involving benign inclusions, an area not well studied to date.

Malignancy within lymph node inclusions should be diagnosed by careful histologic assessment, comparison to concurrent disease if present, and when necessary, diagnostically confirmed by corroborative immunohistochemical studies. Impacts on nodal staging may result in changes to prognosis and management.

## Data Availability

No underlying data was collected or produced in this study.
